# Identification of lncRNA biomarkers for lung cancer through integrative cross-platform data analyses

**DOI:** 10.18632/aging.103496

**Published:** 2020-07-16

**Authors:** Tianying Zhao, Vedbar Singh Khadka, Youping Deng

**Affiliations:** 1 Department of Quantitative Health Sciences, University of Hawaii John A. Burns School of Medicine, The University of Hawaii at Manoa, Honolulu, HI 96813, USA; 2 Department of Molecular Biosciences and Bioengineering, The University of Hawaii at Manoa College of Tropical Agriculture and Human Resources, Agricultural Sciences 218, Honolulu, HI 96822, USA

**Keywords:** lncRNA, lung cancer, biomarker, microarray, RNA-Seq

## Abstract

This study was designed to identify lncRNA biomarker candidates using lung cancer data from RNA-Seq and microarray platforms separately.

Lung cancer datasets were obtained from the Gene Expression Omnibus (GEO, n = 287) and The Cancer Genome Atlas (TCGA, n = 216) repositories, only common lncRNAs were used. Differentially expressed (DE) lncRNAs in tumors with respect to normal were selected from the Affymetrix and TCGA datasets. A training model consisting of the top 20 DE Affymetrix lncRNAs was used for validation in the TCGA and Agilent datasets. A second similar training model was generated using the TCGA dataset.

First, a model using the top 20 DE lncRNAs from Affymetrix for training and validated using TCGA and Agilent, achieved high prediction accuracy for both training (98.5% AUC for Affymetrix) and validation (99.2% AUC for TCGA and 92.8% AUC for Agilent). A similar model using the top 20 DE lncRNAs from TCGA for training and validated using Affymetrix and Agilent, also achieved high prediction accuracy for both training (97.7% AUC for TCGA) and validation (96.5% AUC for Affymetrix and 80.9% AUC for Agilent). Eight lncRNAs were found to be overlapped from these two lists.

## INTRODUCTION

Lung cancer is the leading cause of cancer-related mortality worldwide [1, 2]. According to a report on 2018 global cancer statistics, lung cancer was the most commonly diagnosed cancer in 37 countries, making up to about 11.6% of total cancer cases for both sexes [2]. Based on histology, lung cancer can be divided into two types: small cell lung cancer (SCLC) and non-small cell lung cancer (NSCLC) which account for about 15% and 85% of lung cancer, respectively [3]. NSCLC can be further subdivided into several subtypes: adenocarcinoma (ADC), squamous cell carcinoma (SCC), adenosquamous carcinoma, undifferentiated carcinoma and large cell carcinoma [4]. In 2014, more than 25% of cancer deaths were attributed to NSCLC [5–7]. The overall 5-year survival rate after curative tumor resection is relatively low in lung cancer patients [8] because most already have locally advanced or metastatic disease when diagnosed [9]. Only around 20%-30% of patients have potentially operable, early-stage disease at presentation [9]. Currently, the clinical diagnosis of lung cancer relies mainly on chest X-ray, low dose computed tomography (CT) scans, and other imaging technology which, unfortunately, are encumbered by the harmful effects of radiation and high costs. Although there are invasive methods for auxiliary diagnoses, such as bronchoscopy and biopsy, these methods are painful and time-consuming. Moreover, overlapping symptoms between lung cancer and other chronic respiratory conditions such as cough, dyspnea, chest pain, fatigue, chest infection, hemoptysis, and weight loss often complicate and delay diagnosis [9]. These reasons underscore the important need for non-invasive, sensitive and reliable biomarkers for the early diagnosis of lung cancer.

Advances in high-throughput technologies in recent years have brought a massive increase of multi-omics (e.g. genomics, transcriptomics, proteomics, and metabolomics) data [10]. Potential biomarkers for various cancers have been reported including microRNA for lung cancer prediction and development [5, 11], plasma small ncRNA for early-stage lung adenocarcinoma screening [12], lipid species and seven-gene CpG-island methylation panel for breast cancer diagnosis [13, 14], snail protein for gastric cancer [15], genes and pathways for kidney renal clear cell carcinoma [16], circulatory MALAT1 as a prognostic biomarker for hepatocellular carcinoma [17], and lncRNAs as a breast cancer diagnostic biomarkers for breast cancer [18]. For lung cancer, a panel consisting of SOX2OT, ANRIL, CEA, CYFRA21-1, and SCCA was reported for NSCLC diagnosis, while SOX2OT and ANRIL were described as biomarkers for NSCLC prognosis [19]. Indeed, potential diagnostic and prognostic biomarkers for NSCLC are increasingly being reported such as plasma linc00152 [20], circulating lncRNA PCAT6 [21], AFAP1-AS1 [22], HOTAIR [23, 24], lncRNA 00312 and 00673 [25] for diagnosis, and lncRNA CASC9.5 [26] plus LINC00968 [27] for NSCLC prognosis. In addition, PANDAR [28] and LncRNA RP11 713B9.1 [29] were also described as promising biomarkers and potential therapeutic targets for NSCLC. Patients with NSCLC, advanced nonsquamous NSCLC, and squamous cell histology were suggested to test for EGFR mutations, ALK rearrangements, and ROS1 fusions [30]. These results suggest that multiple biomarker testing may be necessary for lung cancer in the future [30, 31].

Although the central dogma of biology states that the flow of genetic information hardwired in the DNA occurs by transcription into RNA and translation into proteins, non-coding RNAs (ncRNAs) are not translated. The many types of ncRNAs are broadly classified into long and small ncRNAs [32]. In recent years, ncRNAs have been studied as potential biomarkers for diagnosis, prognosis, and subtyping [33]. LncRNAs not only participate in a broad range of biological processes such as cell proliferation, migration, invasion, survival, differentiation, and apoptosis [34] but are also involved in tumorigenesis and metastasis in many cancer types [34–36]. Certain lncRNAs have been proposed as potential biomarkers associated with tumor initiation, progression or prognosis [37]. Indeed, lncRNA discovery is a very active field in cancer biology research [38] and here, we explored the possibility of lncRNAs as potential diagnostic biomarkers in lung cancer through a meta-analysis of publicly available microarray and RNA-Seq data, using integrative cross-platform data analyses, machine learning, and independent validation.

The majority of papers reporting meta-analyses assembled differentially expressed gene (DEG) lists from published experimental studies and then articulated consistently reported DEGs; or integrated multiple datasets from different microarray platforms and then executed statistical tests to discover consistently expressed DEGs [39]. By contrast, our study was designed to test whether microarray and RNA-Seq generate similar results to identify lncRNA biomarkers and whether these two platforms could validate each other. Using data-mining and machine-learning approaches, we identified 8 lncRNAs as potential diagnostic biomarkers. To test the efficiency of the biomarkers of interest, we evaluated and compared their sensitivity and specificity [40]. We also performed function analysis using The Atlas of ncRNA in Cancer (TANRIC) [41], the Database for Annotation, Visualization and Integrated Discovery (DAVID) [42, 43] and Tumor Alterations Relevant for Genomics-driven Therapy (TARGET, accessible at https://software.broadinstitute.org/cancer/cga/target).

## RESULTS

### Combining datasets

**Figure 1:**
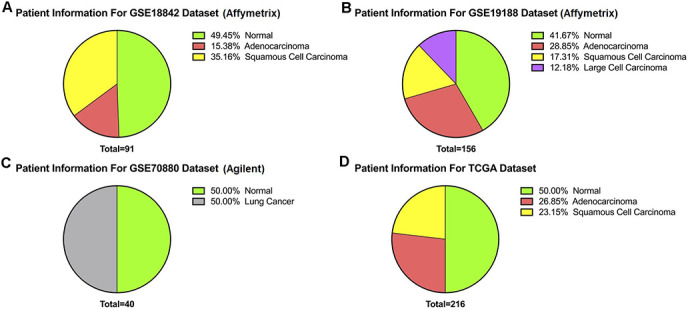
**Patient information.** (**A**) GSE19188 contains 156 samples comprising 65 normal and 91 tumors. (**B**) GSE18842 dataset has 91 samples of which 45 are normal and 46 are tumors. (**C**) Likewise, of 40 samples from GSE70880 20 were normal and 20 were tumor. (**D**) Of 216 samples from TCGA, 108 were normal and 108 were paired adjacent normal.

**Figure 2:**
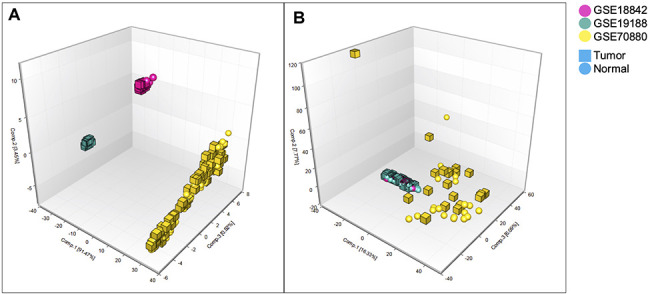
**Principal component analysis 3D plot.** Principal component analysis of GSE18842, GSE19188 and GSE70880 datasets. (**A**) Before normalization, these 3 datasets comprising 399 samples and 963 lncRNAs separated completely. (**B**) After normalization these 3 datasets comprising 287 samples and 963 lncRNAs. GSE19188 and GSE18842 datasets could merge together but GSE70880 was still separate from the other two.

Patient information from the downloaded datasets are summarized in Table 1 and Figure 1: (a) GSE18842 (Affymetrix), (b) GSE19188 (Affymetrix), (c) GSE70880 (Agilent), and (d) TCGA. GSE18842 included 14 adenocarcinoma and 32 squamous cell carcinoma patients, for a total of 46 lung cancer and 45 paired normal samples. GSE19188 included 45 adenocarcinoma, 27 squamous cell carcinoma, and 19 large cell carcinoma patients, for a total of 91 lung cancer and 65 paired normal samples. GSE70880 contained 20 lung cancer samples and 20 paired normal samples from 20 lung cancer patients. The TCGA dataset contained samples from 58 adenocarcinoma patients (116 paired tumor and control) and 50 squamous cell carcinoma patients (100 paired tumor and control) for a total of 216, with 108 normal and 108 paired adjacent normal. Principal component analysis (PCA) performed on the three microarray datasets (GSE18842, GSE19188, and GSE70880) before normalization showed distinct separation of the red, green and yellow components (Figure 2A). After per sample and per gene normalization, PCA revealed that while the two microarray datasets GSE19188 and GSE18842 from the Affymetrix platform could merge well, the GSE70880 dataset from Agilent did not cluster with the Affymetrix ones. As shown in Figure 2B, the red and green components merged together while the yellow one remained separated.

**Table 1:** (A) All datasets patients information.

**Dataset**	**Type**				
	**Adenocarcinoma**	**Squamous-cell carcinoma**	**Large cell carcinoma**	**Total**	**Normal**
**GSE18842**	14	32	0	46	45
**GSE19188**	45	27	19	91	65
**GSE70880**	unknown			20	20
**TCGA**	58	50	0	108	108

**Table:** Table 1. (B) TCGA dataset patients information.

**Tumor subtype**	**Number of patients**
LUAD	44
LUSC	64
**Race**	
Black or African American	8
White	90
Not reported	10
**Age**	
41 - 50	8
51 - 60	14
61 - 70	36
71 - 80	38
81 - 90	12
**Gender**	
Female	32
Male	76
**Number of Samples**	
Healthy	108
Tumor	108

### Identification of most correlated lncRNAs

Based on the PCA, we merged the two Affymetrix datasets, increasing the sample size to a total of 247. We used this merged microarray Affymetrix dataset for training to identify lncRNA biomarkers that are differentially expressed between lung cancer and normal samples, then used the Agilent and TAGA datasets for validation. For the alternative analysis, we used the RNA-seq TCGA dataset, which had a comparable sample size of 216, for training and validated on the Affymetrix and Agilent datasets. The Agilent database was only used for validation in both analysis streams because of its small sample size (n=40). Results of the first analysis where training was done on the Affymetrix dataset using a two-sample t-test are listed in Supplementary Table 1. Correlation Attribute Eval feature selection was performed on a subset of lncRNAs with False Discovery Rate (FDR) adjusted P-value less than 0.05. This process revealed the most related 20 lncRNAs which were: AC008268.1-201, AC027288.3-203, AC146944.4-201, ADAMTS9-AS2-203, AL109741.1-201, AP000866.2-201, CARD8-AS1-201, GATA6-AS1-202, HHIP-AS1-201, HHIP-AS1-203, HSPC324-201, LINC00261-202, LINC01614-201, LINC01852-201, LINC01936-201, LINC02555-201, MAFG-AS1-201, SBF2-AS1-201, TBX5-AS1-201, and TMPO-AS1-202.

Results of the same workflow where the TCGA dataset was used for training are also shown in Supplementary Table 1. Here, two-sample t-test and Correlation Attribute Eval feature selection method revealed the following top 20 lncRNAs: AC004947.1-201, AC007128.1-201, AC008268.1-201, AC023509.2-201, AC087521.1-201, AC107959.1-202, ADAMTS9-AS2-201, ADAMTS9-AS2-203, AP000866.2-201, AP001189.1-201, DDX11-AS1-201, GATA6-AS1-202, HSPC324-201, LINC00163-201, LINC00656-201, LINC01936-201, LINC02016-201, LINC02555-201, TBX5-AS1-201, and VPS9D1-AS1-202.

### Identification of Diagnostic Signature and classifiers

**Figure 3:**
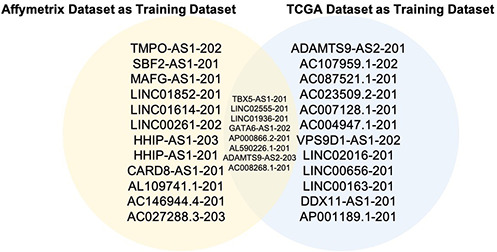
**Overlapped lncRNAs from two top 20 lncRNA lists.** The blue circle stands for the 20 lncRNAs from TCGA as the training dataset, the yellow circle stands for the 20 lncRNAs from the Affymetrix dataset as the training dataset. These 2 lists of 20 lncRNAs have 8 overlapped ones.

As stated above, we first trained on the merged Affymetrix dataset and validated in both Agilent and TCGA datasets. We used the top 20 differential lncRNAs (Figure 3) to build a classification model using the BayesNet algorithm. The training model showed good results – it was able to distinguish cancer from normal samples with a sensitivity of 0.971, specificity of 0.991, and AUC (ROC area) of 0.991 (Table 2). Results of the validation performed on the TCGA and Agilent datasets were as expected (Table 2). Validation performed on the TCGA dataset had a sensitivity of 0.991, a specificity of 0.880 and AUC of 0.992; the Agilent dataset had a sensitivity of 0.850, a specificity of 0.900, and AUC of 0.928.

**Table 2:** Result for affymetrix dataset as training.

**BayesNet**						
**Dataset**	**Sensitivity**	**Specificity**	**AUC**	**Accuracy**	**Precision**	**NPV**
Training-Affymetrix	0.971	0.991	0.990	0.980	0.993	0.965
Validation-TCGA	0.991	0.880	0.992	0.935	0.892	0.990
Validation-Agilent	0.850	0.900	0.928	0.875	0.895	0.857

Similarly, when the top 20 differentiated lncRNAs from training done on the TCGA dataset were used to build a classification model using the Voted Perceptron algorithm, we also achieved very good accuracy in separating cancer from normal samples. The training sensitivity was 0.991, specificity was 0.954 and AUC was 0.995 (Table 3). Validation on the Affymetrix and Agilent datasets also gave the expected results. For the Affymetrix dataset, sensitivity = 0.949, specificity = 0.964 and AUC = 0.965, while for the Agilent dataset, sensitivity = 0.600, specificity = 0.950 and AUC = 0.809. Overall, these results suggest that the lncRNAs used in the models are significantly associated with lung cancer and could be used to discriminate tumors from normal samples.

**Table 3:** Result for TCGA dataset as training.

**Voted perceptron**						
**Dataset**	**Sensitivity**	**Specificity**	**AUC**	**Accuracy**	**PPV**	**NPV**
Training-TCGA	0.944	0.991	0.977	0.968	0.990	0.947
Validation-Affymetrix	0.949	0.964	0.965	0.955	0.970	0.938
Validation-Agilent	0.600	0.950	0.809	0.775	0.923	0.704

**Figure 4:**
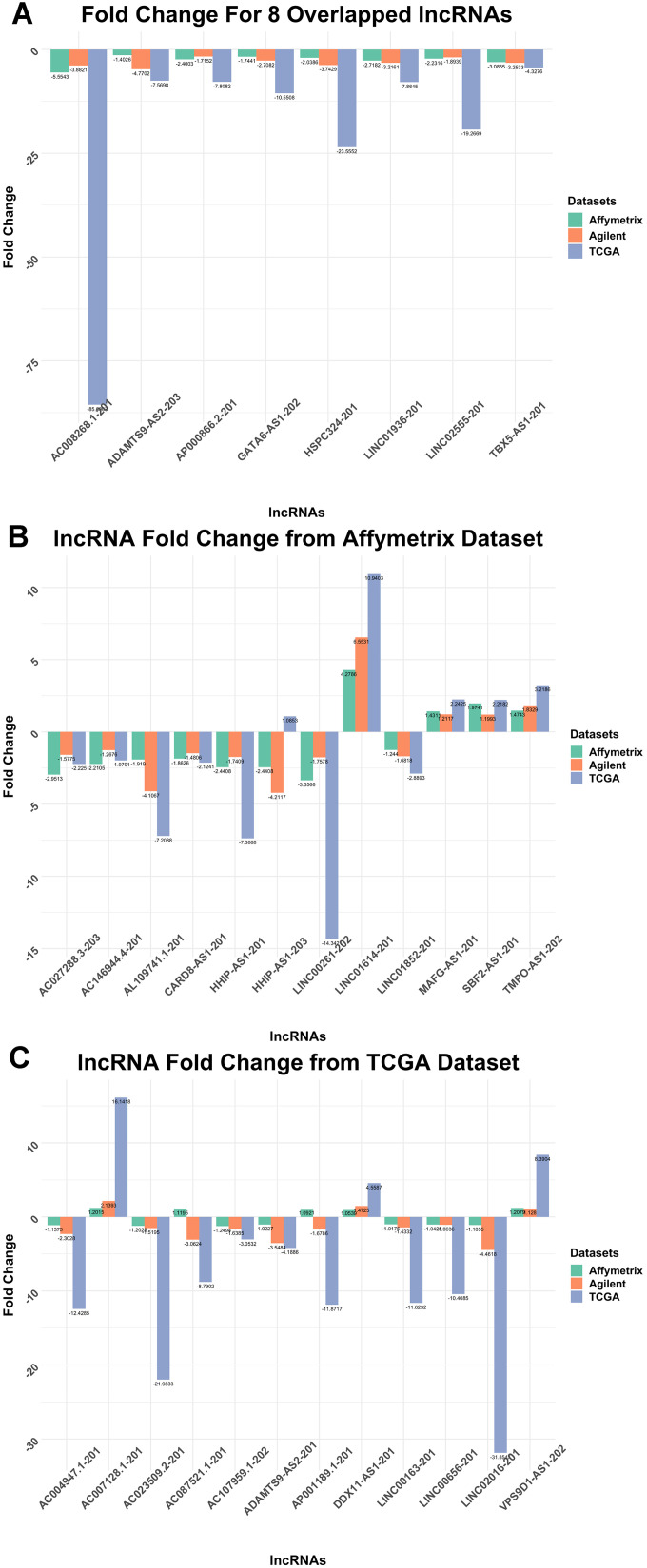
**Fold Change column bar graph.** (**A**) Fold Change for 8 Overlapped lncRNAs T-test result for overlapped 8 lncRNAs. All of them were downregulated. (**B**) lncRNA Fold Change from Affymetrix Dataset T-test result for lncRNAs selected from Affymetrix dataset when it was used as the training dataset. Most of the lncRNAs downregulate, only a few upregulate. (**C**) lncRNA Fold Change from TCGA Dataset T-test result for lncRNAs selected from TCGA dataset as the training dataset. The majority of the lncRNAs downregulate.

**Figure 5:**
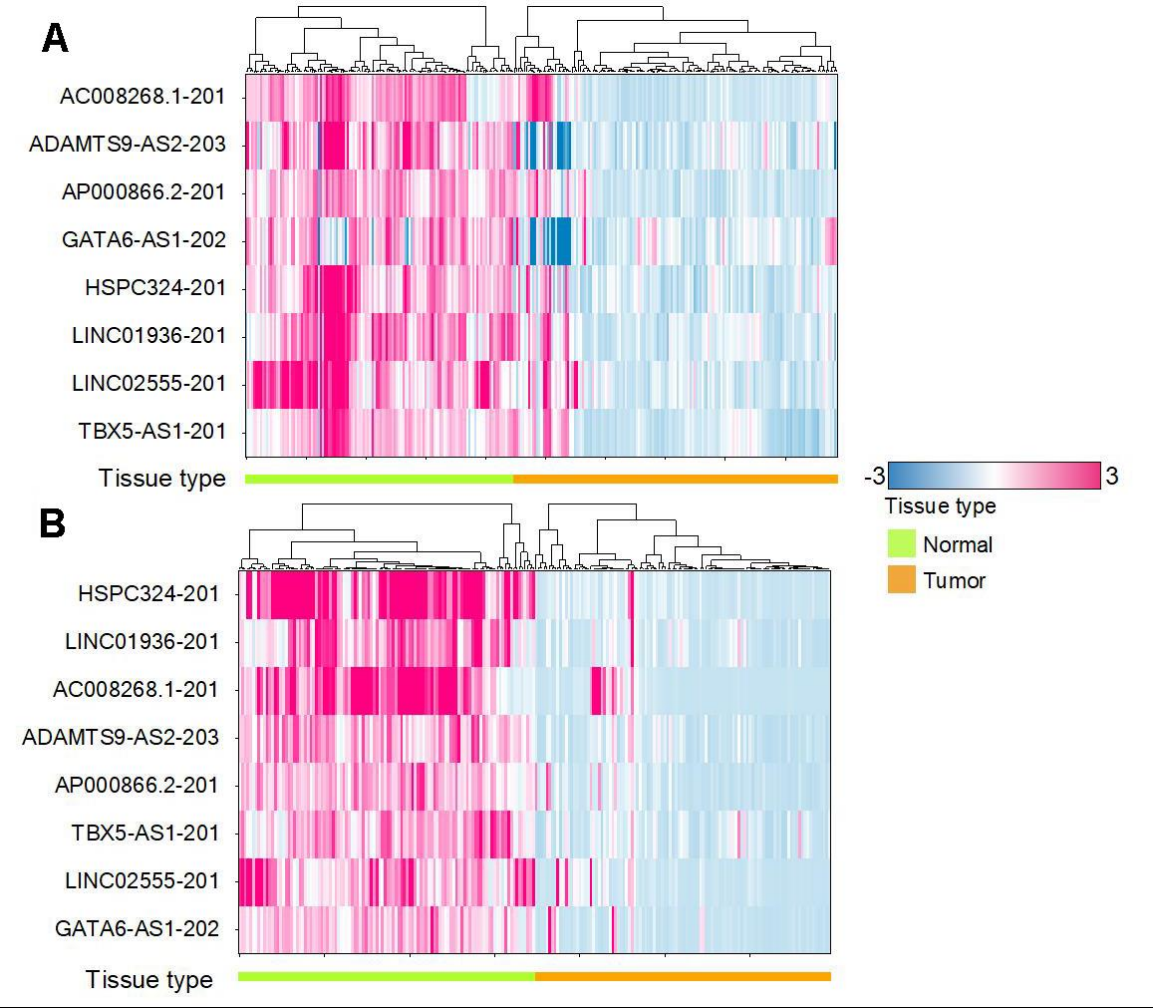
**Hierarchical clustering shows the regulation.** (**A**) Heat map for 8 common lncRNAs in the Microarray dataset. (**B**) Heat map for 8 common lncRNAs in the TCGA dataset. Red means upregulation while blue means downregulation. We can see that all the 8 common lncRNA downregulated in tumor samples.

**Figure 6:**
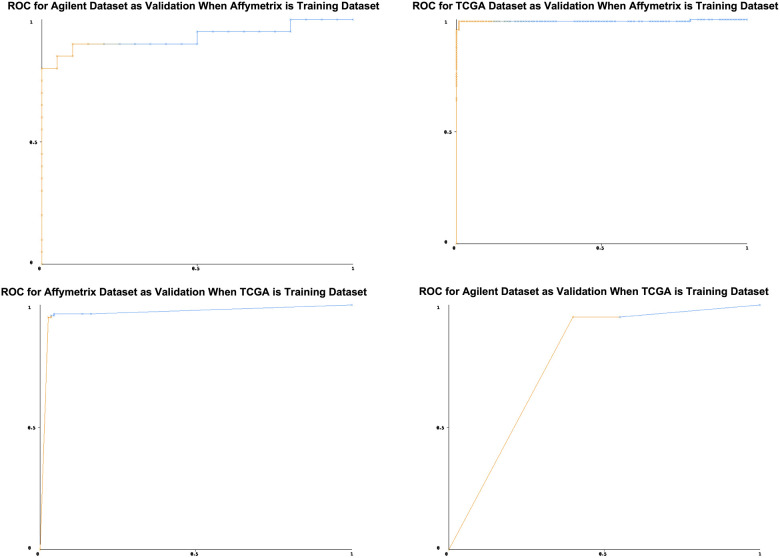
**Receiver Operating Characteristic curves.** The x-axis is the false positive rate, the y-axis is true positive rate. Since the curves are above the diagonal line, this represents good classification results. It means the prediction models can predict lung cancer precisely.

Comparing the top 20 lncRNAs from the Affymetrix and TCGA datasets revealed 8 overlapped lncRNAs (Figure 3) which were all downregulated in cancer (Figures 4, 5). Interestingly, except for a few lncRNAs (i.e., HHP-AS1-203, AC087521.1-201 and AP001189.1-201), the top 20 lncRNAs from the Affymetrix (Figure 4A) or TCGA datasets (Figure 4B), exhibited consistent fold change directions. In fact, all 8 common lncRNAs were downregulated in cancer samples in the three datasets (Figure 4C). By hierarchical clustering, we found that the 8 lncRNAs could completely differentiate the microarray datasets (Figure 5A) and the TCGA dataset (Figure 5B) into normal and tumor groups. Therefore, we used them for further functional analysis. The Receiver Operating Characteristic (ROC) curves illustrate the diagnostic ability of the two models is pretty strong (Figure 6).

### Survival and function analyses

**Figure 7:**
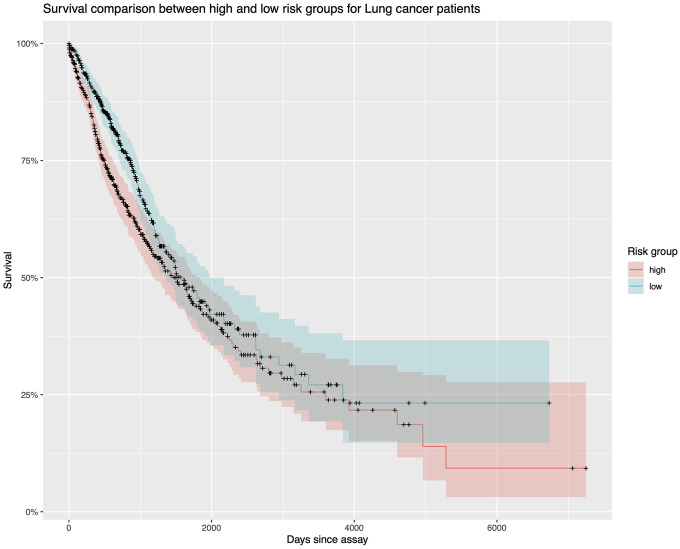
**Survival analysis.** The Kaplan Meier plot for TCGA lung cancer samples. The red line represents those samples with higher lncRNA expression values. The green line represents those samples with lower lncRNA expression levels. Because they intersect several times, the diagnostic ability is not good enough.

We sought to determine if lncRNAs could predict patient survival (Table 4). LINC02555 has p = 0.0299 which shows statistical significance as a prognostic biomarker. However, since the p equals 0.2564 for this cox regression model, there’s no statistical significance between these 8 lncRNAs’ high expression levels and low expression levels. In conclusion, only LINC02555 could be a potential prognostic biomarker. The hazard ratio for LINC02555 is 1.026036, which means that around 1.026036 times as people with higher LINC02555 expression level are dying as people with lower LINC02555 expression level. From the Kaplan Meier plot, we can see that the two lines intersect at some points (Figure 7). This means the prognostic ability is not very good.

**Table 4:** Survival analysis for TCGA lung cancer samples.

**Cox regression result for TCGA lung cancer**					
	**coef**	**Hazard ratio/exp(coef)**	**se(coef)**	**z**	**p**
TBX5-AS1	0.031046	1.031533	0.093602	0.332	0.7401
LINC02555	0.025703	1.026036	0.011835	2.172	0.0299
LINC01936	-0.077035	0.925858	0.109337	-0.705	0.4811
GATA6-AS1	0.010199	1.010251	0.086294	0.118	0.9059
AP000866.2	-0.156948	0.854749	0.118632	-1.323	0.1858
HSPC324	-0.056077	0.945466	0.119202	-0.47	0.638
ADAMTS9-AS2	-0.497076	0.608307	0.608164	-0.817	0.4137
AC008268.1	0.003138	1.003143	0.004481	0.7	0.4838

We also performed functional analysis using the common 8 lncRNAs. This analysis revealed three significant pathways for 3 lncRNAs (Table 5): AC008268.1 in complement and coagulation cascades, ADAMTS9-AS2 in hypertrophic cardiomyopathy (HCM), and TBX5-AS1 in central carbon metabolism in cancer.

**Table 5:** Function analysis.

	**Pathway**	**P-Value**	**Benjamini**
AC008268.1	Complement and coagulation cascades	1.40E-02	7.00E-01
	Protein digestion and absorption	2.70E-02	6.80E-01
	ABC transporters	4.10E-02	6.90E-01
ADAMTS9-AS2	Hypertrophic cardiomyopathy (HCM)	4.70E-02	9.80E-01
	Dilated cardiomyopathy	5.40E-02	9.00E-01
TBX5-AS1	Central carbon metabolism in cancer	4.60E-07	1.30E-05
	Pathways in cancer	2.80E-05	3.90E-04
	ECM-receptor interaction	1.80E-08	4.00E-06
	Focal adhesion	3.90E-08	4.40E-06

## DISCUSSION

Biomarkers from easily accessible tissues such as blood and other body fluids are useful and economical screening tools for various diseases. Such tools are especially important for diseases such as lung cancer where existing diagnostic methods are not able to identify patients at early stages when intervention can be more effective. Indeed, a growing number of studies are using high throughput next-generation sequencing (NGS), especially microarray and RNA-Seq data, to identify diagnostic or prognostic biomarkers for lung cancer [4, 37, 44–48]. Most of these studies examined data from only one technology, either microarray or RNA-Seq.

Microarray and RNA-Seq are two popular ways to measure gene expression. These two technologies have been compared in terms of technical reproducibility, variance structure, absolute expression levels, detection of isoforms, and the ability to identify DEGs and develop predictive models [49]. In general, they are comparable when reporting for high-intensity genes; however, microarrays have been shown to have some systematic biases in their estimation of differential expression for low-intensity genes [50]. Identifying mRNA gene markers for lung cancer has been done by many studies, but very few studies focus on integrative data analysis of lncRNA on lung cancer. This is the reason why we choose lncRNA for the study. Here, through bioinformatics integrative analysis of microarray and RNA-Seq datasets, we identified 8 lncRNAs that could be used as diagnostic or prognostic biomarkers for lung cancer. We used Correlation Attribute Eval feature selection on the statistically significant results to find the top 20 most related lncRNAs. At the same time, we chose the most significant 20 lncRNAs according to their p-values, interestingly, we found that they were exactly the same as what feature selection selected. So, in the case scenario, and the CFS method seems not critical at all. This also means the 20 top ones might truly good ones. We used three microarray datasets from the GEO Repository and an RNA-Seq dataset from TCGA. Of the three microarray datasets, two were on the Affymetrix platform and one on Agilent. Thus, we have datasets from 3 platforms: Agilent, Affymetrix, and TCGA generated using two methods, microarray, and RNA-Seq. We merged the two Affymetrix ones into a combined ‘Affymetrix dataset’ with a sample size of 247. This combined Affymetrix dataset (microarray) and the TCGA dataset (RNA-Seq) were used as training datasets in two separate analysis streams, with the rest of the datasets used for validation.

The top 20 lncRNAs from each training were then used in machine learning to build two training models. We used two classifiers, Voted Perceptron algorithm and Bayes Network learning, for machine learning. All the classifiers were tried in Weka and the best results were chosen for each training dataset. Interestingly, we noted that the best results for the two training datasets were from different classifiers, with Bayes Network learning working better for the Affymetrix dataset and Voted Perceptron algorithm for the TCGA dataset. Overall, we found that the model built from Affymetrix was better than the model from TCGA. We also noted that the Agilent dataset as a validation dataset performed comparatively worse in all models. The sample size of the Agilent dataset was small compared to the others and it did not cluster with the other two datasets when PCA was done after normalization. This could be due to Affymetrix and Agilent are different platforms and the ways for them to design the arrays are not the same, either. Also maybe the Agilent dataset sample size is not big enough. The batch effects even exist for the same platform coming from different labs and more often existed from different platforms. Still, because this study aims to find biomarkers for global lung cancer, we decided to include the Agilent dataset in our analysis.

The training models built separately from the Affymetrix and TCGA datasets and validated on the rest of the datasets resulted in two lists of 20 lncRNAs which include 8 common ones that could be diagnostic lncRNAs. Given that both the sensitivity and specificity are greater than 0.9, we can say that these 8 biomarkers can help predict whether the tissue sample is lung cancer or healthy. Moreover, the good performance of these 8 lncRNA biomarkers strongly suggests that they should work as biomarkers for all lung cancer samples, perhaps including subtypes, although subtypes were not explored in this study. Some of these 8 lncRNAs have previously been described in connection with cell biology and cancer. For example, Qiao et al. reported that TBX5-AS1 was down-regulated in lung cancer tissues compared to non-tumor lung tissues, and its expression was linked to unfavorable prognosis in never-smoking female lung cancer patients [51]. Liu et al. reported that GATA6-AS1 was spatially correlated with the transcription factor GATA6 across the genome [52]. In another study, the long non-coding antisense transcript of GATA6-AS was revealed to interact with epigenetic regulator LOXL2 to regulate endothelial gene expression via changes in histone methylation [53]. Also, Chen et al. found that GATA6-AS1 was down-regulated in LUSC patients and was significantly linked to survival time [54]. ADAMTS9-AS2 was found to correlate with bladder cancer patient survival in an analysis of significantly differentiating RNAs [55] and might play a role in early-stage digit development [56]. In a glioma study, ADAMTS9-AS2 was found to be significantly downregulated in tumor tissues compared with normal ones and reversely associated with tumor grade and prognosis. Their analysis showed that low ADAMTS9-AS2 was an independent predictor of poor survival in glioma [57].

Previous studies have described the function of lncRNAs, but in general, their clinical potential is underexplored [37]. Here, we showed for the first time that lncRNAs are promising biomarkers for the diagnosis of global lung cancer that significantly augment CT imaging which often fails to clearly distinguish between benign and cancer states. By performing in silico analysis on existing normal and tumor tissue samples from GEO NCBI and building prediction models, we identified 8 lncRNAs as promising candidate biomarkers with good diagnostic power based on their high sensitivity and specificity. Our results suggest that in the future, by simply testing the expression level of these 8 lncRNAs in the blood or other body fluids and then generating the prediction model, we may be able to tell if there is lung cancer or not. This will be especially useful because, in the clinic, patients prefer non-invasive detection methods like blood tests rather than invasive methods like a biopsy. As such, it is important that these biomarker candidates be experimentally validated in the laboratory using body fluid samples. We are hopeful that if validated in blood samples, we may be able to create a simple blood test to diagnose lung cancer.

Our study is significant because it reports promising biomarker candidates with solid cross-validation bioinformatics data analysis on different platforms of a pretty large sample size. These biomarkers could be tested in blood serum or plasma samples in future studies. Besides providing potential novel diagnostic biomarkers for lung cancer, our study also provides novel candidate molecules and pathways for mechanistic studies on lung cancer development and carcinogenesis and for the development of new targets for lung cancer treatment.

## CONCLUSIONS

We identified 8 lncRNAs as potential diagnostic biomarkers for NSCLC through integrative cross-platform data analyses. This data mining and machine learning approach would be an efficient and economical screening method for tumor biomarker discovery. Moreover, we are now in an exciting time in bioinformatics when both high-throughput tools and data are increasingly accessible for tumor biomarker discovery. Our study can also help understand the development of lung cancer and provide potential novel targets for lung cancer treatment.

## MATERIALS AND METHODS

### Overview of the workflow

**Figure 8:**
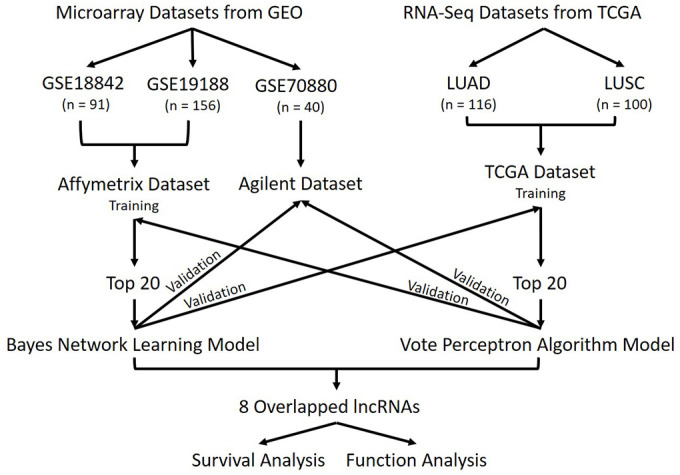
**Workflow.** Schematic overview of this study. Three datasets were downloaded from GEO, they are GSE18842, GSE19188, and GSE70880. A total of 287 samples were in GEO datasets. Two datasets were downloaded from TCGA, including the LUAD dataset and the LUSC dataset. Totally 216 samples were contained in those LUAD and LUSC datasets and we combined them as TCGA datasets. Datasets were divided into 3 groups based on their platforms. Affymetrix dataset and TCGA dataset were used as training sets separately then validated using the other datasets. The lncRNAs in common were used for survival analysis and functional analysis.

To detect lncRNAs differentially expressed between healthy and lung cancer tissues, we employed a one-factor (cancer/normal) experimental design in which datasets containing lung cancer samples and adjacent normal tissue samples were selected (Figure 8). This approach narrows the variation of data and allowed sufficient statistical power. Based on this design, we downloaded three lung cancer microarray datasets, GSE19188 [58], GSE18842 [59] and GSE70880 [44, 60], with a total of 287 samples, from the GEO repository, and 216 RNA-Seq samples from TCGA (http://cancergenome.nih.gov/). For the datasets from array-based platforms, GSE19188 and GSE18842 were combined as Affymetrix dataset and GSE70880 was named as Agilent dataset.

LncRNA names were obtained from BioMart and a total of 963 lncRNAs common to every dataset were identified for further analyses. At first, we used the Affymetrix dataset for training. Differentially expressed lncRNAs at FDR adjusted p-value by the Benjamini-Hochberg procedure less than 0.05 using Student’s t-tests were selected. We uploaded the data to Weka (version 3-8-2) [61], then used correlation Attribute Eval feature selection to get the most statistically significant related lncRNAs. We selected the top 20 differentially expressed lncRNAs to build a model using Bayes Network learning, then performed validation on the TCGA and Agilent datasets.

Next, we used the TCGA dataset for training and selected differentially expressed lncRNAs at FDR adjusted p-value less than 0.05 using Student’s t-tests. We applied Correlation Attribute Eval feature selection on the statistically significant results to find the most related lncRNAs and used the top 20 to build the model using the Voted Perceptron algorithm incorporated in Weka. This time, validation was performed on the Affymetrix and Agilent datasets. Of the top related 40 lncRNAs from the two analyses, we identified 8 overlapping lncRNAs. These were further interrogated for survival and function analysis.

### The cancer genome atlas (TCGA) datasets as RNA sequencing (RNA-seq) dataset

Lung adenocarcinoma (LUAD) and lung squamous cell carcinoma (LUSC) datasets from TCGA incorporating RNA-Seq FPKM data from 216 lung tissue samples and matched adjacent normal tissue samples were downloaded. The annotations of the TCGA dataset were acquired from BioMart in R (version 3.4.3).

### Gene expression omnibus (GEO) datasets as microarray dataset

We conducted a search of the GEO Microarray database using keywords “ncRNA, lung cancer and RNA seq*” to find microarray datasets. We also searched for papers in PubMed that were related to lncRNA, RNA sequencing and lung cancer. We only downloaded microarray datasets with at least 20 lung cancer samples and adjacent normal tissue samples because studies with a smaller sample size would be challenging to merge due to batch effects. The downloaded GEO datasets are: GSE70880, GSE19188, GSE18842. GSE19188 and GSE18842 contain NSCLC samples but the lung cancer types in GSE70880 are unknown. The annotations of each dataset were obtained from BioMart in R (version 3.4.3). The TCGA or GEO datasets were merged based on transcript names and a total of 963 lncRNAs common in all the datasets were included for further analyses.

### Data normalization for GEO datasets

First, we used per sample every sample median value across all the lncRNAs in GSE19188, GSE70880, and GSE18842 datasets. Then we performed per gene normalization based on every lncRNA expression median value across all the samples in these three microarray datasets. We used RBoxPlot in Array Studio software to check the normalization results. LncRNAs in the GSE70880 dataset with missing data were excluded. Principal component analysis based on the common 963 lncRNAs was done on the combined datasets. GSE19188 and GSE18842, both from the Affymetrix platform, clustered well, but GSE70880 from the Agilent platform could not cluster with the other two. So, GSE19188 and GSE18842 were combined and named as Affymetrix dataset and GSE70880 was named Agilent dataset for further analyses. RBoxPlot were obtained using Array Studio 10 (Supplementary Figure 1)

### Data normalization for TCGA dataset

The lung cancer RNA-seq data from TCGA was normalized based on the Fragments Per Kilobase of transcript per Million mapped reads (FPKM). The FPKM data from TCGA were log 2 transformed after adding 0.1.

### Screening for differentially expressed lncRNAs

For each dataset, the difference in expression of lncRNAs between cancer and normal was examined by a two-sample t-test. The fold change and regulation direction were then reported. Each of the datasets was tested for differential expression by a two-sample t-test using Array Studio 10. Statistically significant differentially expressed lncRNAs were selected with a False Discovery Rate (FDR) adjusted p-value less than 0.05 and fold change greater than 1.3 in at least one dataset.

### Training datasets and validation

The Affymetrix dataset containing differentially expressed lncRNAs was used as a training dataset first. Feature selection correlation method was done using Weka (version 3.8.2) and the top 20 lncRNAs were selected for further analysis. Hierarchical cluster analysis was performed using Array Studio 10. Bayesian network classifier (BayesNet) was used on the top 20 Affymetrix lncRNAs and validated in TCGA and Agilent datasets. Sensitivity and specificity were calculated based on the Bayesian network results. The formulas for sensitivity and specificity are:

Sensitivity (True Positive Rate) = True Positive / (True Positive + False Negative);

Specificity (True Negative Rate) = True Negative / (True Negative + False Positive).

Likewise, the TCGA dataset with differentially expressed lncRNAs was used as a training dataset and Weka (version 3.8.2) feature selection correlation method was used to identify the top 20 lncRNAs. Voted Perceptron was used for classification, and then validated in both Affymetrix and Agilent datasets separately. Sensitivity and specificity were calculated based on Voted Perceptron results. Subsequently, hierarchical cluster analysis was performed using Array Studio to check the expression levels of the eight overlapping lncRNAs. The Receiver Operating Characteristic (ROC) curves were plotted using Weka software.

### Survival analysis

Cox regression analysis was performed using the survival package in R. The lncRNAs with a p-value of less than 0.05 were considered associated with survival. This analysis includes all lung cancer samples with survival information available from TCGA.

### Function analysis

Overlapping lncRNAs from the top 20 lncRNAs obtained after feature selection from Affymetrix and TCGA datasets respectively, were used for functional analysis.

We used TANRIC to find correlated mRNA, miRNA, protein and somatic mutation with the common lncRNAs in LUSC and LUAD datasets, respectively. The lists of correlated genes were then used in TARGET and DAVID for functional analysis.

## Supplementary Material

Supplementary Figure 1

Supplementary Table 1
