# Supplementary material for: Identification of lncRNA biomarkers for lung cancer through integrative cross-platform data analyses
Source: Aging (Albany NY). 2020 Jul 16;12(14):14506–27. doi: 10.18632/aging.103496 (PMC7425463; doi:10.18632/aging.103496)
Supplement: Supplementary Figure 1 [file aging-12-103496-s002..pdf]

SUPPLEMENTARY FIGURE

GSE18842 Raw Data (Affymetrix Dataset, n = 91)

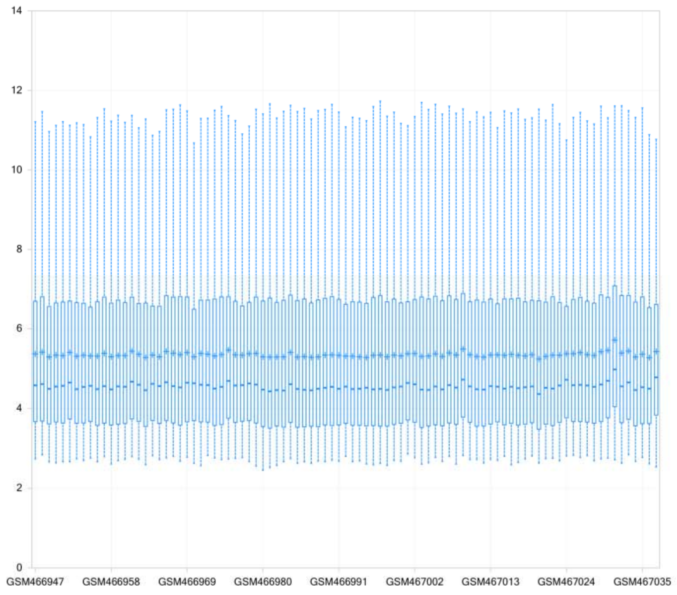

GSE19188 Raw Data (Affymetrix Dataset, n = 156)

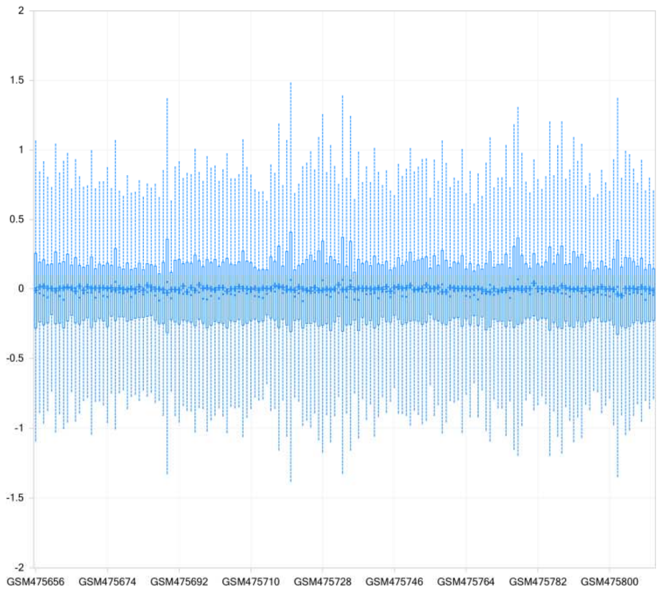

GSE70880 Raw Data (Agilent Dataset, n = 40)

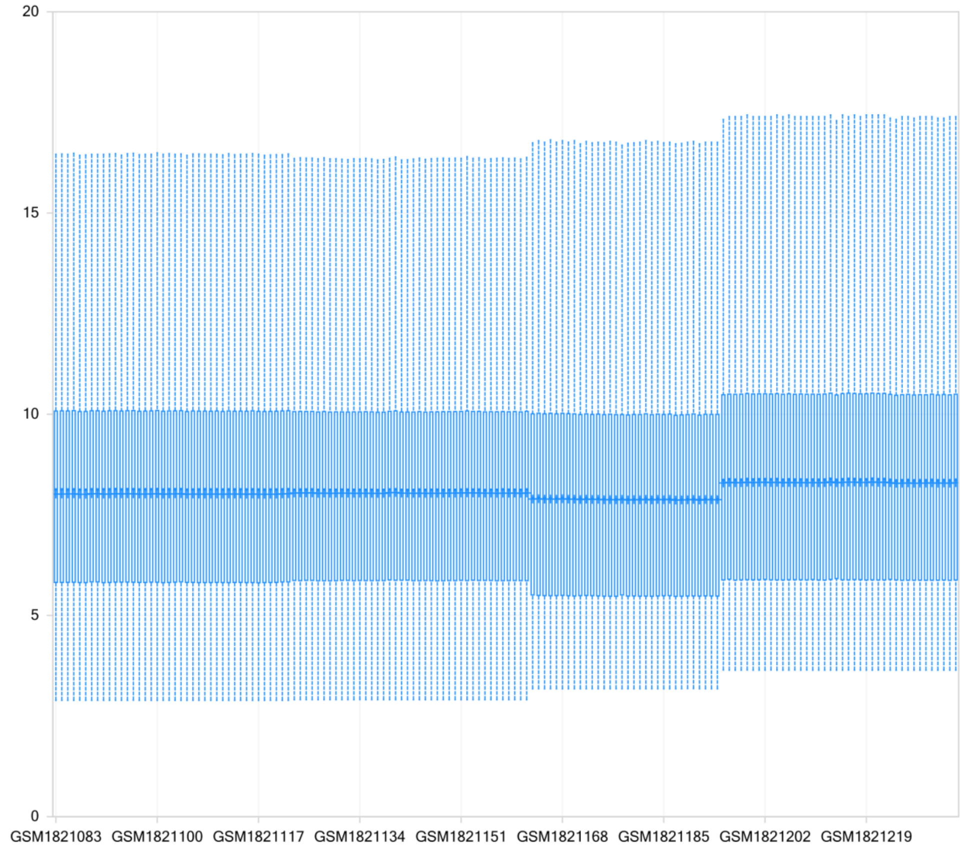

TCGA Raw Data (n = 216)

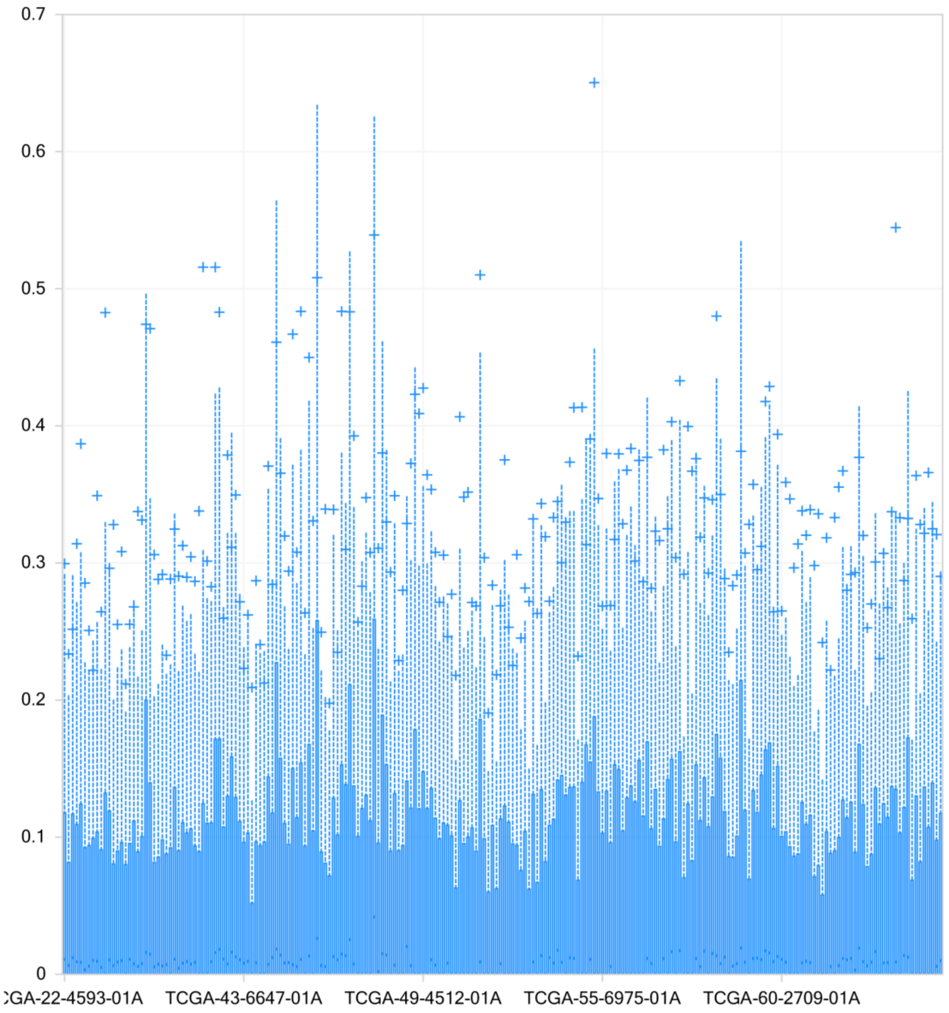

GSE18842 Normalized Data (Affymetrix Dataset, n = 91)

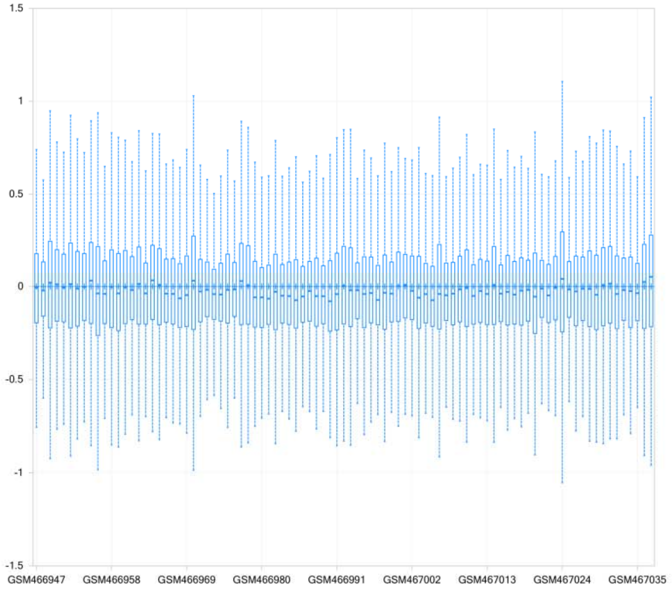

GSE19188 Normalized Data (Affymetrix Dataset, n = 156)

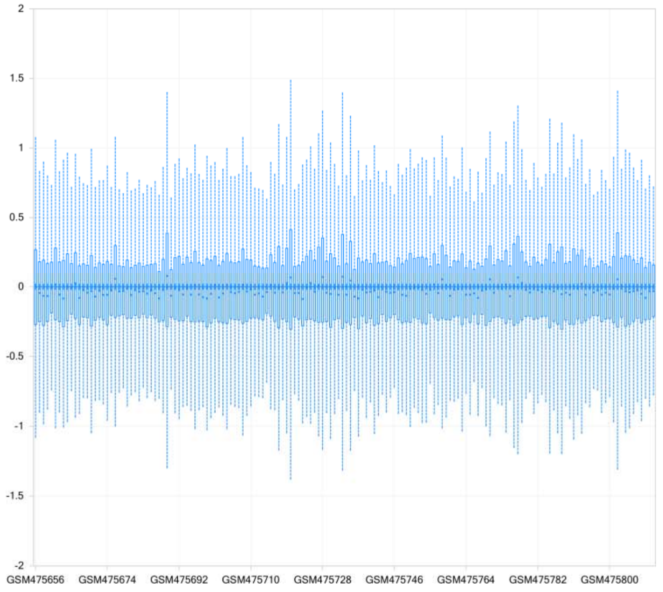

GSE70880 Normalized Data (Agilent Dataset, n = 40)

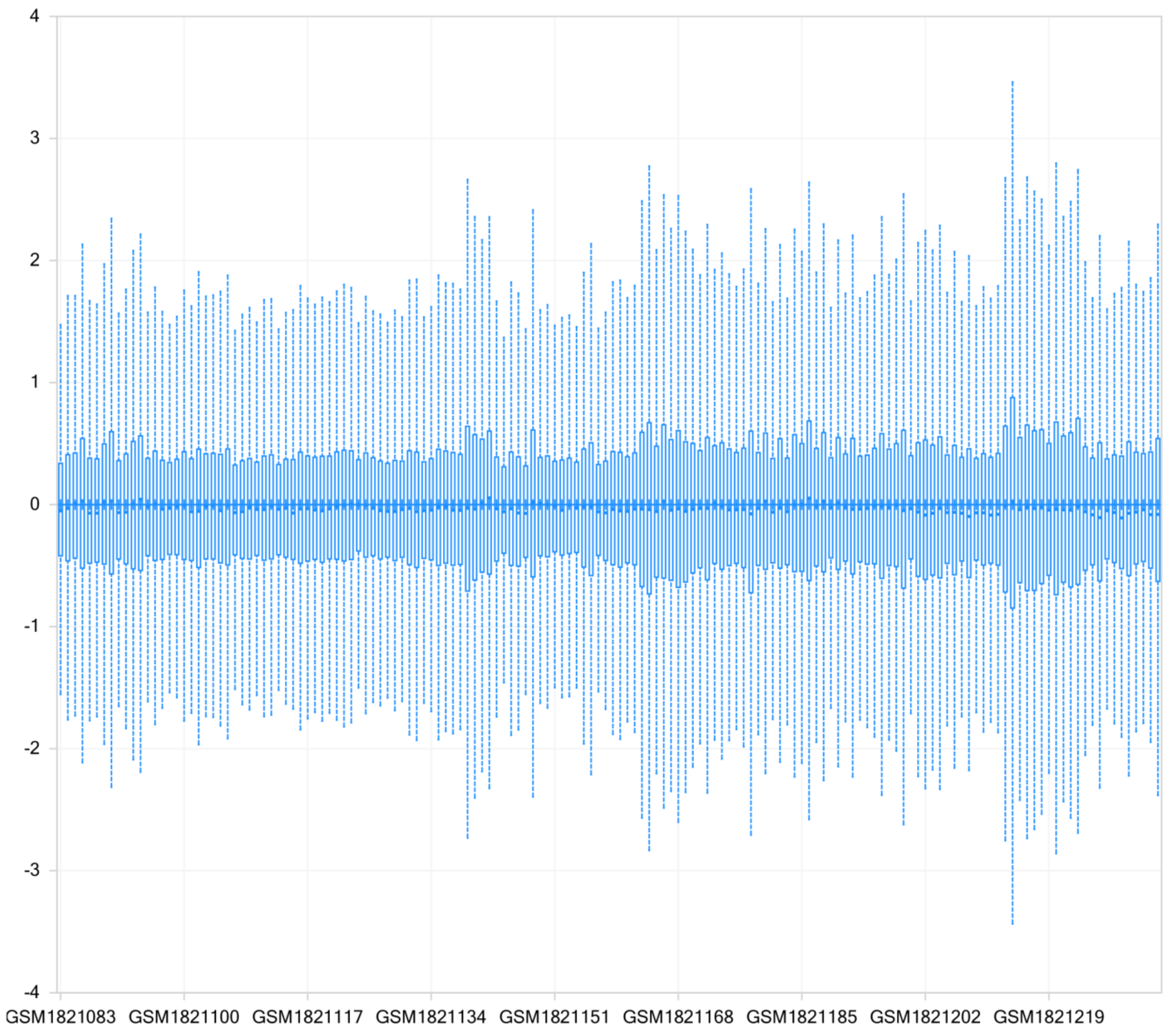

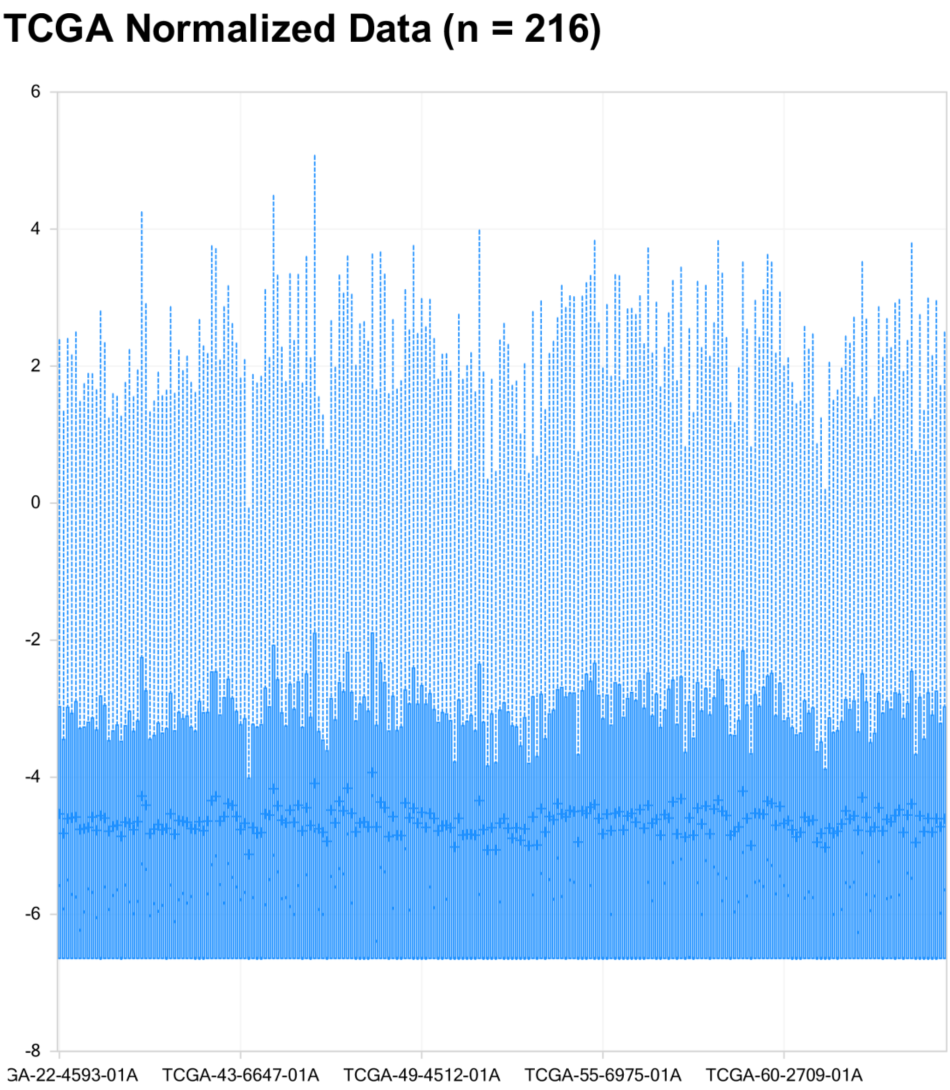

Supplementary Figure 1. The boxplots of GSE18842 Raw Data, GSE19188 Raw Data, GSE70880 Raw Data, and TCGA Raw Data were plotted. In the boxplot for GSE18842 raw data, we can see that its mean is not center to zero. The mean for GSE19188 raw data is centered on zero. The mean for GSE70880 raw data is neither center to zero, nor consistent. Some samples from GSE70880 has a lower mean than others. The mean for TCGA raw data is not consistent, either. Normalized data for every dataset were plotted. For GSE18842, GSE19188, and GSE70880, we centered their mean values to zero and removed their batch effects. For the TCGA dataset, we made the mean on the same level.
